# Walking through Architectural Spaces: The Impact of Interior Forms on Human Brain Dynamics

**DOI:** 10.3389/fnhum.2017.00477

**Published:** 2017-09-27

**Authors:** Maryam Banaei, Javad Hatami, Abbas Yazdanfar, Klaus Gramann

**Affiliations:** ^1^School of Architecture and Environmental Design, Iran University of Science and Technology, Tehran, Iran; ^2^Department of Psychology, University of Tehran, Tehran, Iran; ^3^Department of Psychology and Ergonomics, Berlin Institute of Technology, Berlin, Germany; ^4^Center for Advanced Neurological Engineering, University of California, San Diego, La Jolla, CA, United States; ^5^School of Software, University of Technology Sydney, Sydney, NSW, Australia

**Keywords:** neuroarchitecture, EEG, mobile brain/body imaging (MoBI), architectural interior form, virtual reality, HMD

## Abstract

Neuroarchitecture uses neuroscientific tools to better understand architectural design and its impact on human perception and subjective experience. The form or shape of the built environment is fundamental to architectural design, but not many studies have shown the impact of different forms on the inhabitants’ emotions. This study investigated the neurophysiological correlates of different interior forms on the perceivers’ affective state and the accompanying brain activity. To understand the impact of naturalistic three-dimensional (3D) architectural forms, it is essential to perceive forms from different perspectives. We computed clusters of form features extracted from pictures of residential interiors and constructed exemplary 3D room models based on and representing different formal clusters. To investigate human brain activity during 3D perception of architectural spaces, we used a mobile brain/body imaging (MoBI) approach recording the electroencephalogram (EEG) of participants while they naturally walk through different interior forms in virtual reality (VR). The results revealed a strong impact of curvature geometries on activity in the anterior cingulate cortex (ACC). Theta band activity in ACC correlated with specific feature types (*r*_s_ (14) = 0.525, *p* = 0.037) and geometry (*r*_s_ (14) = −0.579, *p* = 0.019), providing evidence for a role of this structure in processing architectural features beyond their emotional impact. The posterior cingulate cortex and the occipital lobe were involved in the perception of different room perspectives during the stroll through the rooms. This study sheds new light on the use of mobile EEG and VR in architectural studies and provides the opportunity to study human brain dynamics in participants that actively explore and realistically experience architectural spaces.

## Introduction

In recent years, advancements in neuroscientific methods have made it possible to study the influence of different architectural styles on human perception and affective states. The field of neuroarchitecture studies the effects of the built environment on its inhabitants by using neuroscientific tools (Edelstein, 2008; Nanda et al., 2013). Historically, architectural studies were based on philosophical constructs or analysis of behavioral patterns to relate human responses to the design under investigation (Edelstein and Macagno, 2012, p. 28). While such approaches provide descriptive evidences, they cannot clearly specify the reasons for different behaviors in built environments. Of late, neuroscientific studies have been attempting to fill the gap between architecture and psychology by describing some of the underlying mechanisms that explain how differences in architectural features cause behavioral outcomes (Vartanian et al., 2013). Several neuroarchitectural studies have investigated different architectural styles (Choo et al., 2017), embodiment (Vecchiato et al., 2015), contours (Vartanian et al., 2013), height and enclosure (Vartanian et al., 2015), built vs. natural environment (Roe et al., 2013; Banaei et al., 2015), lighting (Shin et al., 2014), color (Küller et al., 2009), or the impact of the built environment on human memory (Sternberg, 2010, p. 147).

The present study focused on the form of interior places and its impact on the human perceiver. The Oxford Dictionary defines “form” as “the visible aspect of a thing; the shape or figure of the body as distinguished from the face” (OED, 2016). The form is one of the main aspects in architectural design and deciding which forms to use is one of the most challenging aspects of the design process (Madani Nejad, 2007, p. 3). While architects are responsible for considering the function and technology of their design, they are mostly free in designing the form according to their personality and individual preferences (Ackerman et al., 2017). To find a more objective approach to describe forms and their impact on the inhabitant, this study built a framework to design forms based on a data-driven approach using cluster analysis of form features and neuroscientific methods to evaluate the impact of form on human perceivers. In this “neuroscience of the experience of architecture” (Robinson and Pallasmaa, 2015, p. 82) study, we focused on the emotional impact of different interior forms on inhabitants, as affective responses toward environments are accrued automatically and unconsciously (Coburn et al., 2017).

A few studies investigated the relationship between architectural form and emotion (Bar and Neta, 2006; Madani Nejad, 2007; Shemesh et al., 2017). Some of these studies sought to define curved lines with adjectives like “serene”, “graceful”, and “tender-sentimental”, and described angles as “robust”, “vigorous” and “somewhat more dignified” (Lundholm, 1921; Poffenberger and Barrows, 1924; Hevner, 1935). Roelfsema et al. (1999) concluded that having too many curved forms could cause stress. Neuroscientific approaches using imaging methods like functional magnetic resonance imaging (fMRI) showed that people preferred curved interior forms over rectilinear forms and that perception of the former was associated with increased activity within the anterior cingulate cortex (ACC; Vartanian et al., 2013). The authors further demonstrated the involvement of anterior mid cingulate cortex in approach-avoidance decisions for enclosed rooms (Vartanian et al., 2015). Other fMRI studies investigating the relationship of object forms with emotional experience demonstrated the activation of the amygdala by sharp contoured objects (Bar and Neta, 2007; Ghoshal et al., 2016, p. 102). The parahippocampal place area (PPA) and the lateral occipital complex (LOC) play a general role in the perception of architectural styles (Choo et al., 2017). While the PPA is also active for shapes with rectilinear features (Nasr et al., 2014), the LOC is activated by shapes with curved features (Nanda et al., 2013) and the representation of object identity and location (Cichy et al., 2011). Moreover, visual cortex areas such as V1, V2, V3 and V3a are involved in three-dimensional (3D) shape perception (Welchman et al., 2005). However, despite the wealth of new insights from these imaging approaches, none of the studies used a quantitative method to describe forms and their role in architectural design. Most studies used only rectilinear and curvature forms as differentiating aspects (Dazkir, 2009; Nanda et al., 2013; Vartanian et al., 2013; Nasr et al., 2014).

According to a previous study, forms of built places are more complicated and contain more form features than curvature and rectilinear geometries (Banaei et al., 2017). A systematic analysis of living room interiors showed that real places combine different form features with different densities, and that the combination of different forms significantly shapes the interior. Cluster analysis of different forms revealed the main form features as linear solids, curved linear solids, and surfaces and rectangular Euclidean solids with different angles, locations, and scales as these play an important role in feature distinction. Most rooms contained rectangular forms, and the results revealed that linear solids were important for distinguishing between different rooms. The reality of built environments reveals a mixture of different forms. To understand the functional role of forms for the design process, we need to understand not only the effects of isolated forms on the perceiver, but also the impact of their combination. Therefore, this study used a combination of forms derived from formal cluster analysis of real built environments.

Besides a reduction to only a few architectural forms, almost all previous studies investigated the effect of forms on the perceiver using two-dimensional (2D) images. This stands in marked contrast to human experiences of architectural spaces in the real world, which is inherently 3D (Coburn et al., 2017). Recently, virtual reality (VR) technologies like CAVE environments were introduced to study the perception of 3D forms (Welchman et al., 2005). However, while the use of 3D stimulus material allowing rotation of the head and torso is a significant progress toward more realistic measures, these studies lack natural movement through the built environment (Vecchiato et al., 2015). It is essential to look around and see the places from different perspectives to allow a natural perception of the 3D environment. This is especially the case for the perception of 3D shapes. Just by navigating through the environment, our visual system can learn about our surroundings (Eagleman, 2015, p. 47). Movement is essential for an embodied perception of 3D environments (Gramann, 2013), hence the impact of architectural design on the moving inhabitant. Therefore, in this study, we allowed natural movement through the built environment using head-mounted VR displays (HMD VR) allowing exploration of 3D architectural spaces. The VR was combined with mobile brain/body imaging (MoBI) synchronously recording movement and brain dynamics during active exploration of the virtual environment (Gramann et al., 2014). To overcome the restrictions of traditional electroencephalography (EEG) studies, MoBI synchronizes recordings of brain dynamics in actively behaving participants with motion capture and other data streams, and uses data-driven analysis approaches to dissociate brain from non-brain sources of activity that underlie cognitive and affective changes during natural movement in the environment (Makeig et al., 2009; Gramann et al., 2010a, 2011; Gwin et al., 2010; Gwin, 2012; Jungnickel and Gramann, 2016).

Combining MoBI with VR allows researchers to study the designed environment and its impact on human experience even before construction. Moreover, investigating the brain dynamics using EEG provides unobtrusive insights into the cognitive and affective processing of the human in the environment with adequate temporal resolution. Although fMRI and functional near-infrared spectroscopy (fNIRS) provide a spatial resolution to describe the brain areas involved in the processing of the architectural and urban design (Tsunetsugu et al., 2005; Vecchiato et al., 2015; Choo et al., 2017), these methods lack the high temporal resolution that cognitive processes reveal.

Since natural perception of the built environment is based on active movement leading to different 3D perspectives of the same, the aim of this study was to investigate the effects of different interior form features on human brain activity in participants naturally exploring the 3D space. Based on the results of previous imaging studies and previous subjective emotional ratings of materials used in our study, we expected significant differences in the perception of different architectural forms in brain regions relevant to shape processing and brain areas related to affective processes.

## Materials and Methods

### Participants

Data was collected from 17 healthy right-handed volunteers with a mean age of 28.6 years (σ = 2.6). Two subjects were removed from the analysis because of technical problems during the EEG recordings and all reported results are based on the final group of 15 participants (8 females, 7 males). As architects and non-architects would have different reactions towards space (Kirk et al., 2009), we chose participants without prior education in architectural studies. All participants had normal or corrected to normal vision and none reported a history of neurological disease. Volunteers were compensated 10 Euro/h or received course credit for their participation. All participants provided written informed consent before the experiment and the study was approved by the local ethics committee of the Technische Universität Berlin according to the guidelines of the German Psychological Society.

### Experimental Design and Procedure

Based on the results of the previous study (Banaei et al., 2017), 25 different form clusters were extracted from 343 interior images of living rooms from different architectural epochs with varying architectural styles. The formal clusters, derived from high dimensional clustering based on internal and external similarity functions, led to different descriptive form features. Features’ dimensions are 8 types, 13 geometries, 6 scales, 5 locations and 6 angles (Banaei et al., 2017). We chose the top five features of each cluster to build 3D models of rooms using Autodesk’s 3Ds Max-student version (San Francisco, CA, USA) replicating the clusters’ density of form features. Virtual rooms (W × L × H: 5.0 × 7.5 × 3.0 m) were built with white interior color and identical lighting. The only difference between the rooms was their form features. The unity game engine software (Unity Technologies, San Francisco, CA, USA)^1^ was used to create the rooms and the tests in VR.

In the first study, we used a virtual self-assessment manikin (SAM; Bradley and Lang, 1994) test to select a subset of clusters for the EEG study. Seventy-five rooms were constructed with three rooms for each of the 25 clusters to represent rooms with form differences. Forty volunteers with a mean age of 27.6 years (σ = 4.7) participated in the study wearing a head-mounted display (HMD-Gear VR) to evaluate the 75 rooms with respect to their emotional impact. Participants provided their responses while standing. One participant was removed from the study because of technical issues resulting in data from 21 female and 18 male participants.

The obtained SAM ratings represented the subjective experience for each of the 75 rooms on the pleasure, arousal, and dominance scale. The scores provided information about the subjective experience of pleasure (+P) vs. displeasure (−P), arousal (+A) vs. non-arousal (−A), and dominance (+D) vs. submissiveness (−D). The 2P × 2A × 2D emotion categories of Russell and Mehrabian’s PAD emotion model were used to divide rooms into different emotional groups (Mehrabian and Russell, 1974). To this end, each room was assigned to an emotional group according to its positive or negative rate in the pleasure, arousal, and dominance scales. The average scores for each dimension and room were computed and rooms with similar emotional ratings were grouped together. The results divided the 25 formal clusters into five groups that differed along the emotional model dimensions (+P +A −D, −P −A −D, −P +A −D, +P +A +D, and +P −A +D). To test whether the rooms in the different groups were significantly different in their emotional responses, three mixed measure ANOVAs with a 3 (rooms representing a cluster) × 5 (emotional groups) design for each of the dependent variables pleasure, arousal, and dominance were computed using Greenhouse-Geisser corrected values for the results with violations of sphericity. The results showed a significant main effect of the emotional groups on pleasure scores (*F*_(2.055,78.092)_ = 29.992; *p* < 0.001; *η*^2^ = 0.441), arousal scores (*F*_(2.227,84.636)_ = 11.577; *p* < 0.001; *η*^2^ = 0.234), and the dominance scores (*F*_(2.207,83.857)_ = 18.732; *p* < 0.001; *η*^2^ = 0.330). None of the analyses revealed a significant impact of the rooms or an interaction effect (all *p*s ≥ 0.131). Subsequently, the eight lowest ranked clusters were removed, resulting in a selection of 17 from the initial 25 clusters that used for further analysis. Besides these 17 clusters, one room was added to the study containing a simple cubic room without significant form features serving as comparison room.

**Figure 1:**
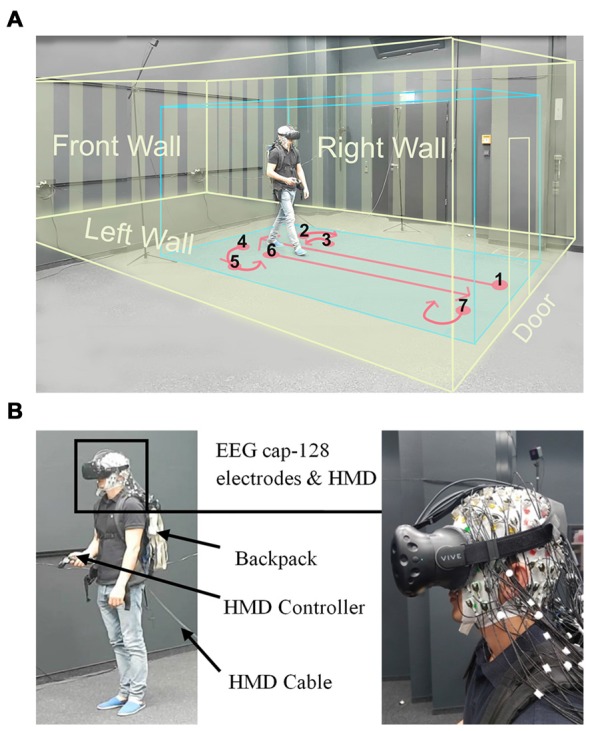
Experimental setup. **(A)** Displays a participant while walking inside the laboratory wearing a head-mounted VR display (HTC Vive), the virtual room, and an electroencephalogram (EEG) cap. The participant provided written consent that the video recordings may be used in scientific publications. The blue box displays the borders of the walking space and the yellow box indicates the dimensions of the virtual room. The instructed walking paths are marked in red and all the walls and the door of virtual room are displayed in yellow. **(B)** Left: displays a participant wearing the head-mounted display (HMD; HTC Vive) and holding a controller in his hand. The EEG transmission systems were located in a backpack. The HMD cable was connected to the rendering PC and was extended to allow free movement of participants, Right: close view on the HMD place on an electrode cap with 128 actively amplified electrodes.

In the following EEG study, for each of the selected 17 clusters, one additional room was constructed to increase the number of rooms to be tested in the EEG experiment. This resulted in four rooms with comparable feature densities, each for the 17 clusters of interest plus one room without features for comparison. This led to an overall number of 69 rooms presented in separate trials. Participants walked inside the different rooms with the HMD (HTC Vive) and an EEG cap (see Figure 1). After each room trial, the same virtual SAM test that was used in the first study was recorded. The SAM ratings were used as a factor for the later EEG analysis. Finally, the Stroop test (Stroop, 1935) was recorded at the end of each room trial to test whether the participants demonstrated comparable levels of attention and involvement with the task for all rooms.

### EEG Recording

The EEG was recorded from 128 active electrodes referenced to an electrode near CP1 with a sampling rate of 1000 Hz and band-pass filter from 0.016 Hz to 250 Hz (BrainAmps and Move System, Brain Products, Gilching, Germany). Electrodes were placed in an elastic cap (EASYCAP, Herrsching, Germany) using a custom layout with equidistant distribution of electrodes approximating the 5% system (Oostenveld and Praamstra, 2001). Electrode locations were recorded using an optical tracking system (Polaris Vicra, NDI, Waterloo, ON, Canada). Electrode impedance was kept below 15 kOhm in all electrodes.

### Motion Capture Recording

Two HTC Vive lighthouse cameras captured the movement of participants with a sampling rate of 75 Hz. The data stream containing the location (x, y and z), angles (x, y, z and w) and all the events of the virtual rooms and tests were produced by unity software (Unity Technologies, San Francisco, CA, USA) custom functions. All data streams, namely EEG, motion, and events from the experimental protocol were synchronized and recorded using the Lab Streaming Layer Software (Kothe, 2014).

### Procedure

**Figure 2:**
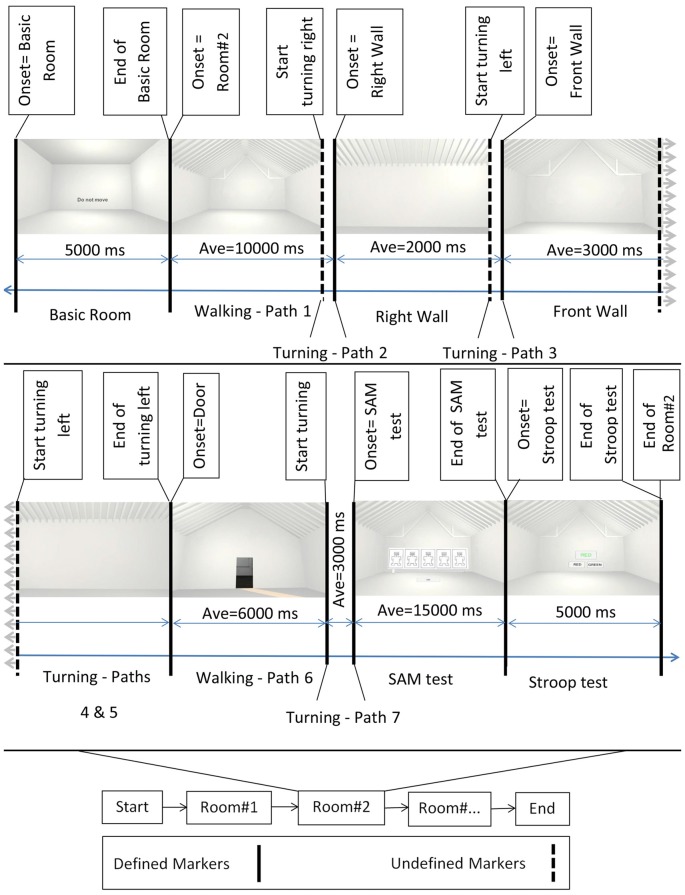
Block diagram of the experiment. Bottom middle: the onset of the different rooms. Top: example of rooms’ perspectives as participants perceived them while walking. First and then second row from left to right, after showing the Basic room for 5000 ms with a “Do Not Move” sign, the trial started and participants walked inside the virtual room. They then turned 90 degrees to their right and faced the *right wall*. Subsequently they turned 90 degrees to their left and faced the *front wall*, followed by an additional turn of 90 degrees to their left to face the *left wall*. After one additional turn of 90 degrees to their left, participants faced the entrance *door*. Then, participants walked back to the starting point (entrance door) and turned 180 degrees to face the room. At the end of each trial, the virtual self-assessment manikin (SAM) test and the Stroop test were shown. Defined markers describe events in the experimental protocol that were controlled and appeared at predefined time points during the experiment. These included, e.g., the onset of the basic room, the onset of the SAM test, etc. Undefined markers denote events that were not determined by the experimental protocol, i.e., the time point of some of the turnings depended on the individual preferred movement speed and viewing time. Sample room was created from Banaei et al. (2017).

After reading the instructions, participants entered a VR demonstration of how to use the HMD controller to answer the virtual questions and how to walk in VR, as shown in Figure 2. After about 10 min of practice, participants proceeded with the EEG recordings.

An experimental trial started with a *basic room* (complete cubic shape), with participants receiving an instruction not to move (a “Do Not Move” sign at eye level). The basic room was displayed for 5 s, followed by the presentation of one of the 69 experimental rooms. After the onset of the experimental room, participants were instructed to walk inside the room until they reached the border of the virtual room. They then turned 90 degrees to their right and faced the *right wall*. Afterward, they turned 90 degrees to their left and faced the *front wall*, followed by an additional turn of 90 degrees to the left to face the *left wall*. After one additional turn of 90 degrees to their left, the participants faced the entrance *door*. At that point, the participants walked back to the entrance door and turned 180 degrees to face the room again (see Figure 1). Participants were then presented with a virtual SAM test and had time to rate the room according to the pleasure, arousal, and dominance scale. The SAM test was followed by the two-color (red and green) virtual Stroop test, and participants had 5 s to provide as many correct answers as possible. After the Stroop test, the basic room with the “Do Not Move” sign appeared again for 5 s initiating the next trial. The experiment comprised four randomized blocks of 18 room trials each (17 clusters of interest plus one room without features) that each room contain three perspective trials. At the end of the second and fourth blocks, a sign (“The End”) was displayed, meaning that the participants have a 10-min break or the end of experiment, respectively. After the experiment, the participants answered questions regarding their VR experience and their health conditions after the experiment.

### EEG Analysis

#### EEG Data Preprocessing

The analysis was done using open source EEGLAB toolbox^2^ (Delorme and Makeig, 2004) and custom MATLAB functions (The Mathworks, Inc., Natick, MA, USA). The data was filtered using a high-pass filter (1 Hz) followed by a low-pass filter (100 Hz) and subsequent down sampling to 500 Hz. Periods in the raw signal containing artifacts were removed manually. Eye movements were not regarded as an artifact. Noisy channels were removed manually by visual inspection of the data and EEGLAB automatic channel rejection. On average, 99.3 EEG channels remained for further analyses (range: 80–122; σ = 14.04).

Independent component analysis (ICA) was computed using the EEGLAB runica function and the data was re-referenced to an average reference after ICA analysis. ICA analysis was used in mobile EEG studies to separate brain and non-brain activities (Gwin et al., 2010; Gramann et al., 2011, 2014). An equivalent dipole model was calculated for each IC using a boundary element head model (BEM) based on the MNI brain (Montreal Neurological Institute, MNI, Montreal, QC, Canada) as implemented by DIPFIT routines (Oostenveld and Oostendorp, 2002). Each model was aligned by adjusting its individually measured landmarks (nasion, inion, vertex and preauricular points) with the landmarks as implemented in the head model. ICs representing brain activity were selected from the overall 1490 ICs based on their spectra, location of their equivalent dipole model and the residual variance (<15%) of the corresponding dipole models. Dipoles placed outside of the head model were not further considered. In total, 305 ICs remained for all participants for further analysis with an average of 20.3 ICs per subject (range: 12–29, σ = 5.7).

The continuous EEG data was epoched into 1.2 s long epochs with the onset of events indicating different views into the rooms selecting the room onset, the front wall, and the right wall, including a 0.5 s pre-stimulus baseline. For a majority of participants, the instructed left turn to face the left wall did not allow to extract any peaks in quaternion numbers because it was indistinguishable from an ongoing 180 degree turn to the left. Although participants were instructed to wait for some seconds before turning towards the entrance, most participants did not wait long enough for meaningful epoch extraction. Thus, the analyses focused on three room perspectives including the room onset, the right wall, and the front wall. The average number of perspective trials for each room’s perspective were 59.73 (σ: 11.14), 37.53 (σ: 13.37) and 56.2 (σ: 14.81) for room onset, right wall and front wall, respectively.

By using the EEGLAB pre-clustering function, distances between all ICs were calculated with the weighted measures of ERP, power spectrum, event-related spectral perturbations (ERSPs), inter-trial coherences (ITCs), the components’ scalp maps, and their equivalent dipole model locations. Power spectrum was computed with fast fourier transform (FFT) and frequencies between 3–75 Hz were used for clustering. ERSP and ITC were calculated with 200 time points and 3-cycle wavelets (with a Hanning-tapered window applied). Principal component analysis (PCA) reduced the dimensions of all measures to the first 10 principle components, except dipole location with three dimensions. Measures were normalized, weighted, and combined into cluster position vectors. All the measures were weighted with the standard weighting of 1, except dipole locations (*w* = 25) and ERSP (*w* = 10) like in similar MoBI studies (Jungnickel and Gramann, 2016). Clustering was done by a K-means algorithm in EEGLAB with the number of clusters set to 24. ICs with a distance higher than 3 SDs to the mean of any cluster centroid were set as an outlier resulting in seven outlier ICs. In case clusters contained more than one IC from one participant, extra ICs were moved to the outlier cluster (53 ICs). The dipole location of cluster centroids were estimated based on the Talairach^3^ software (Lancaster et al., 2000), providing approximate locations of the cluster centroids. Clusters including ICs from at least 60% of all participants were selected and ERSP results are reported for these clusters.

### Motion Capture Analysis

**Figure 3:**
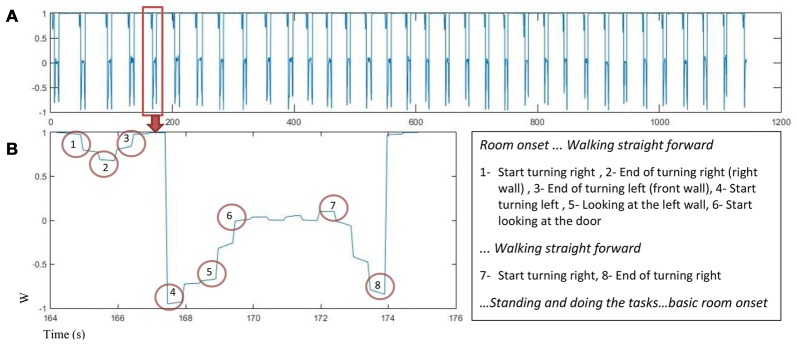
The quaternion number changes as a function of time for one participant **(A)** *w* value for 34 rooms in about 1200 s. **(B)** Left: *w* changes in one room while participant is walking and turning, the turning points are marked, Right: turns marker description.

Motion capture data was used to find turning points in the trial sequence for each participant. The quaternion number (w) was analyzed to find the turning points using the peaks in the quaternion chart as an indicator. Figure 3 displays changes in quaternion numbers for a representative walking path of one participant moving through the 34 rooms (A) with a closer look on the path for one room (B).

## Results

The participants’ presence in VR was measured after the experiment with a questionnaire in 9-point Likert scale (−4 to 4). Analyzing the result with a one-sample *t*-test (*p* < 0.05) showed that the virtual environment appeared real to the participants ($\bar{x}$ = 1.07; σ = 1.38). Participants had the feeling that the virtual environment surrounded them ($\bar{x}$ = 1.14; σ = 1.23) and they did not have the feeling to see only images ($\bar{x}$ = −0.78; σ = 1.52). Furthermore, the virtual world did not seem more real than the real world ($\bar{x}$ = −1.57; σ = 1.55) and participants felt presence in virtual space ($\bar{x}$ = 1, σ = 1.24). In summary, the data revealed that the designed virtual environment was adequate to support a feeling of being in a real environment.

### SAM Ratings

The virtual SAM test was used to assess the experienced emotional impact of the formal clusters (17 clusters) to be used for further EEG analyses. Participants rated each room according to the pleasure, arousal, and dominance scale after strolling through the rooms. The mean of the three scales for each formal cluster was calculated and formal clusters with similar emotional categories were grouped based on the 2P × 2A × 2D emotion categories of Mehrabian and Russell (1974) PAD emotion model (see “Experimental Design and Procedure” Section). The results revealed two groups of +P −A +D (Group 1) and +P +A +D (Group 2). Ten of the 17 formal clusters were categorized as Group 1 and seven formal clusters as Group 2. The emotional categories of the simple cubic comparison room was +P −A +D (pleasure: $\bar{x}$ = 5.05, σ = 1.72, arousal: $\bar{x}$ = 3.93, σ = 2.25, and dominance: $\bar{x}$ = 5.80, σ = 2.10) which was sorted into Group 1. As this room was not rated as emotionally neutral, we did not use it as a baseline for the EEG analyses. Three mixed measure ANOVAs with a 4 (rooms per cluster) × 2 (emotional rating groups) design for each of the dependent variables pleasure, arousal, and dominance were computed to test for the significant differences between the two groups. The Greenhouse-Geisser correction was used wherever there was a violation of sphericity. The results revealed a significant differences in the two emotion categories for pleasure scores (*F*_(1,14)_ = 10.692; *p* = 0.006; *η*^2^ = 0.433) and arousal scores (*F*_(1,14)_ = 8.423; *p* = 0.012; *η*^2^ = 0.376). There was no significant effect of grouping for the dominance scale (*p* = 0.128) and no significant effect of rooms per cluster for pleasure, arousal, and dominance scale (all *p*s ≥ 0.055). Also, there was no interaction effect for all three ANOVAs (all *p*s ≥ 0.307). The *post hoc* test showed that Group 2 had higher scores in both pleasure and arousal scales (*p* = 0.006). A Spearman’s rank-order correlation was calculated to assess the relationship between SAM ratings in the first and the main study. The results revealed significant positive correlations between SAM ratings in the two studies for the pleasure scale (*r*_s_ (17) = 0.621, *p* = 0.008) and the arousal scale (*r*_s_ (17) = 0.687, *p* = 0.002). There was no significant correlation of ratings in the dominance scale between the two studies (*p* = 0.075). Based on the missing differences in the dominance ratings, we focused on the pleasure and arousal dimensions for further analysis.

### Form Features

According to the SAM scales, 17 formal clusters were divided into two groups differing in the pleasure and the arousal scale. Each form feature defines different aspects of the space providing a location, scale, angle, type and geometry (Banaei et al., 2017). As an example, consider a simple cube (type) with Euclidian geometry placed in an empty room; the room itself consists of six surfaces (type) and 12 edges (type) that have one-dimensional (1D) linear and 2D rectangular geometry with orthogonal angles. The room will appear differently if the cube is positioned on the central wall as compared to the ceiling (location) and whether the cube is small or big (scale). The cube’s angle can be towards or against the basic room XYZ-axis.

**Figure 4:**
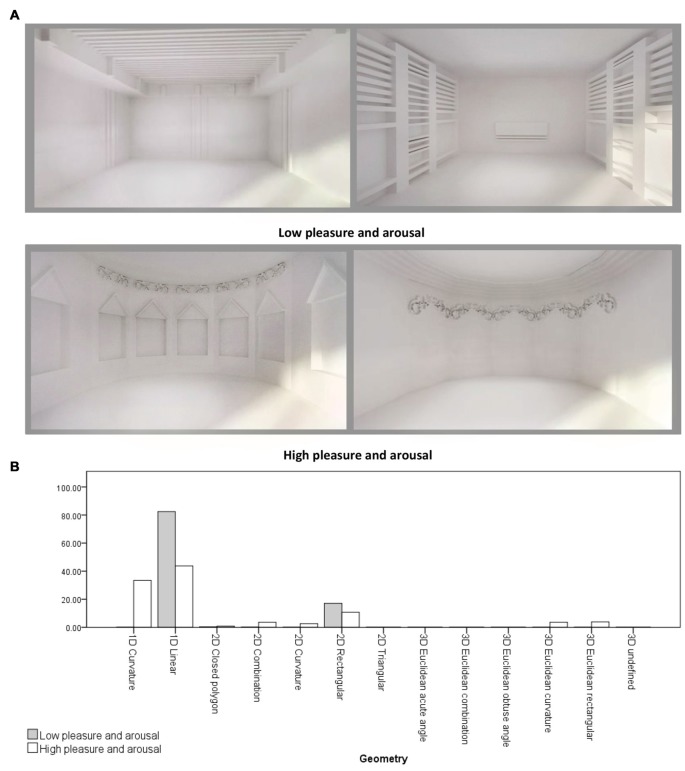
**(A)** Samples of virtual rooms created by form features, 1st row: two rooms belong to low pleasure and arousal group, 2nd row: two rooms belong to high pleasure and arousal group. **(B)** Geometry differences between two emotion rating groups. Virtual rooms were created from Banaei et al. (2017).

An independent-samples *t*-test was conducted to compare the form features’ density in the two emotion rating groups (Group 1: Low pleasure and arousal and Group 2: high pleasure and arousal) regarding location, object to object scale, object to context scale, Z-axis angle, XY-axis angle, type and geometry. There was a significant differences in 1D linear geometries between Group 1 ($\bar{x}$ = 0.82, σ = 0.12) and Group 2 ($\bar{x}$ = 0.44, σ = 0.36; *t*_(6.909)_ = 2.70, *p* = 0.031). Also, there were significant differences in the sum of linear geometry densities (1D, 2D and 3D) between Group 1 ($\bar{x}$ = 0.99, σ = 0.009) and Group 2 ($\bar{x}$ = 0.58, σ = 0.40; *t*_(6.004)_ = 2.74, *p* = 0.034), and in the sum of curvature geometry densities (1D, 2D and 3D) between Group 1 ($\bar{x}$ = 0.001, σ = 0.003) and Group 2 ($\bar{x}$ = 0.43, σ = 0.43; *t*_(6)_ = −2.62, *p* = 0.039). Therefore, the main differences between the two groups were in the linear and curvature geometries (see Figure 4).

### SAM Rating and Form Features Correlation

A Spearman’s rank-order correlation was computed to assess the relationship between arousal scale and form features. The results revealed positive correlations between arousal and curved Z-axis angle, sum of Z-axis of curved, sloped and combination angles, 1D curvature geometry, and sum of curvature geometries ((*r*_s_ (18) = 0.471, *p* = 0.049), (*r*_s_ (18) = 0.584, *p* = 0.011), (*r*_s_ (18) = 0.657, *p* = 0.003) and (*r*_s_ (18) = 0.703, *p* = 0.001), respectively). In addition, negative correlations were observed between arousal and angles towards XY-axis, sum of zero and straight Z-axis angles, full object to context scale, and sum of linear geometries ((*r*_s_ (18) = −0.482, *p* = 0.043), (*r*_s_ (18) = −0.499, *p* = 0.035), (*r*_s_ (18) = −0.562, *p* = 0.015) and (*r*_s_ (18) = −0.628, *p* = 0.005), respectively).

Moreover, a Spearman’s rank-order correlation for the covariation of pleasure scale and form features demonstrated positive correlations between pleasure and curved Z-axis angle, sum of Z-axis of curved, sloped, and combination angles, neutral object to object scale, and sum of curved geometries ((*r*_s_ (18) = 0.543, *p* = 0.020), (*r*_s_ (18) = 0.683, *p* = 0.002), (*r*_s_ (18) = 0.554, *p* = 0.017) and (*r*_s_ (18) = 0.713, *p* = 0.001), respectively). The results further demonstrated negative correlations between pleasure and angles towards XY-axis, zero Z-axis, and sum of Z-axis of zero and straight, floor type, horizontal object to object scale, and sum of linear geometries ((*r*_s_ (18) = −0.618, *p* = 0.006), (*r*_s_ (18) = −0.516, *p* = 0.028), (*r*_s_ (18) = −0.654, *p* = 0.003), (*r*_s_ (18) = −0.492, *p* = 0.038), (*r*_s_ (18) = −0.487, *p* = 0.040) and (*r*_s_ (18) = −0.739, *p* = 0.0004), respectively).

### Stroop Test

Analyzing the Classic Stroop test at the end of each room trial revealed no performance differences between the formal clusters. A repeated measures ANOVA for correct to total Stroop answers (C/T) also revealed no significant differences between the formal clusters (*F*_(17,238)_ = 0.944; *p* = 0.523; *η*^2^ = 0.063). Therefore, it can be concluded that the attention of participants was comparable for the different rooms and cannot serve as explanation for potential differences in the EEG data. The average ratio of C/T was 0.24 (σ = 0.03). We found one participant with less attention in the Stroop test (participant #8, $\bar{x}$ = 0.19). We removed 16 room trials (formal clusters: cl3, cl5, cl8, cl13) in which the participant revealed a C/T below the individual standard deviation ($\bar{x}$ = 0, 0.11, 0.09, 0.11, respectively in formal clusters) of that participant from the further analysis.

### EEG Data

**Figure 5:**
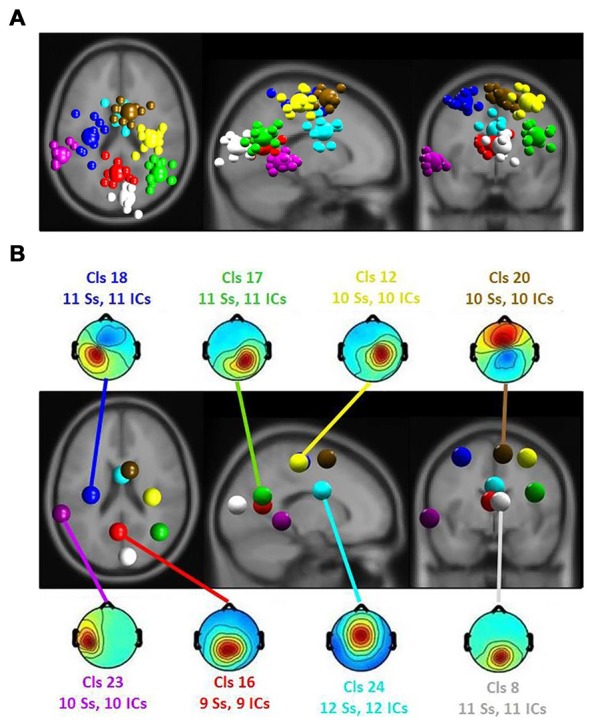
**(A)** Equivalent-dipole locations of main clusters’ independent components (ICs; small spheres) and their centroids (larger spheres), projected to the horizontal, sagittal and coronal views of the standard Montreal Neurological Institute (MNI) brain. **(B)** Clusters’ centroids and their scalp maps, marked with cluster number (Cls #), number of participants (# Ss) and number of ICs (# ICs) for each cluster in the corresponding color.

Repeated measures ANOVA were computed for the ERSP in using a 2 (emotion rating) × 3 (room perspective) study design. To evaluate multiple comparisons, the significance level (*p* value) was corrected using the FDR procedure (Benjamini and Hochberg, 1995). Only clusters with ICs of at least 60 percent of all participants were selected for analyses (see Figure 5).

**Figure 6:**
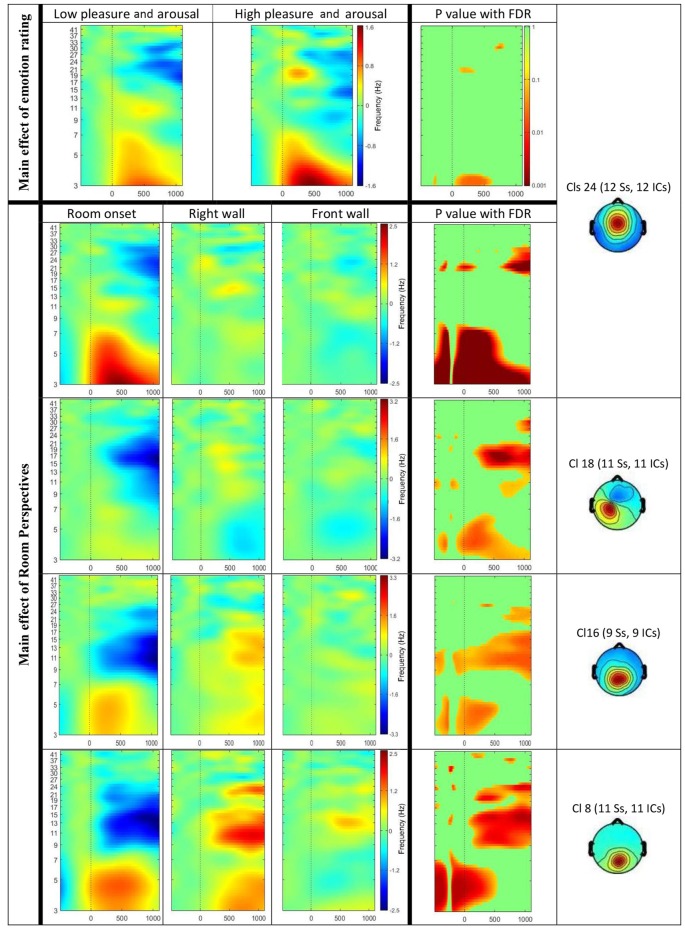
Event-related spectral perturbation (ERSP) results for the main effect of visual sequences (Top) and emotion rating (Bottom). The Y-axis shows frequency (Hz) from 3 Hz to 41 Hz and the X-axis shows time (ms) from −500 ms to 1200 ms. Significant results displayed for ERSPs in or near the anterior cingulate cortex (ACC; Cls 24), the left precentral gyrus (Cls 18), the posterior cingulate cortex (Cls 16) and the occipital lobe (Cls 8). Top row displays differences in ERSP dependent on the emotional ratings for the lower pleasure and arousal group compared to the higher pleasure and arousal group. The room perspectives are displayed in the order starting with room onset, followed by onset of the right wall and the front wall. Significant differences are marked with exact *p* values with FDR correction in the most right columns.

The ERSP results revealed a significant main effect of the affective ratings (*p* < 0.05) and a main effect of the room perspective (*p* < 0.05), but no interaction effect (*p* > 0.05; see Figure 6). Multiple clusters demonstrated pronounced differences in several frequency bands for different room perspectives. Significant differences between the room perspectives were observed in or near the occipital lobe (Cls 8, *x* = 6, *y* = −84, *z* = 17, Brodmann area (BA) 18), in or near the precentral gyrus in both hemispheres (Cls 12, *x* = 32, *y* = −20, *z* = 53, BA 4 and Cls 18, *x* = −32, *y* = −18, *z* = 54, BA 4), the posterior cingulate cortex (Cls 16, *x* = −3, *y* = −58, *z* = 16, BA 23), the middle temporal gyrus in both hemispheres (Cls 17, *x* = 40, *y* = −58, *z* = 24, BA 39 and Cls 23, *x* = −63, *y* = −40, *z* = 0), and the superior frontal gyrus (Cls 20, *x* = 9, *y* = 7, *z* = 56, BA 6). There were significant main effects of the different room perspectives in or near the ACC (Cls 24), the left precentral gyrus (Cls 18), the posterior cingulate cortex (Cls 16) and occipital lobe (Cls 8). The ACC (Cls 24) mainly revealed theta band modulations from 3 Hz to 7 Hz in the time window up to 1200 ms after stimulus onset and in the beta band between 20 Hz–30 Hz for the time period from 700 ms to 1200 ms post stimulus. Spectral perturbations in or near the left precentral gyrus (Cls 18) revealed a significant effect in the theta band between 3 Hz and 5 Hz from stimulus onset to 500 ms post stimulus and for the frequency band from 14 to 20 Hz from 250 ms to 1200 ms post stimulus. The posterior cingulate cortex (Cls 16) demonstrated variations in the theta band around 3–5 Hz from stimulus onset to approximately 500 ms after stimulus onset with additional spectral perturbations in the frequency ranges between 9–17 Hz for the time period 300–1200 ms post stimulus. The occipital lobe (Cls 8) mainly revealed differences in theta band perturbations between 3 Hz and7 Hz for the first 500 ms and modulations of the alpha (8–13 Hz) and the beta (13–30 Hz) frequency bands from 200 ms to 1200 ms after stimulus onset. There were no further significant effects for any of the clusters in the ERSP analyses.

In addition, significant differences in specific frequency bands for the two valence groups were observed in or near the ACC (Cls 24, *x* = 0, *y* = 1, *z* = 26, BA 24). The analysis of theta activity (3–7 Hz) originating in or near the ACC (Cls 24) revealed a significant main effect for the emotional ratings with higher activity in the group with higher pleasure and arousal ratings. As Figure 6 shows, the difference was observed mainly between 100 ms to 600 ms post stimulus onset in the frequency band 3–4 Hz.

### ERSP and Form Features Correlation

The cluster with its centroid located in or near the ACC (Cls 24) was the only cluster demonstrating differences in power modulations dependent on room perspectives as well as valence ratings. Thus, follow up analyses investigated the relationship of spectral perturbations in this cluster with architectural features as well as valence ratings. Spearman’s rank-order correlations were computed to assess the relationship between ACC changes and form features. There was a positive correlation between theta power from 3 Hz to 3.5 Hz from 150 ms to 500 ms originating from the ACC and sum of Z-axis curved, sloped, and combination angles, surfaces with attachment type, 1D curvature geometry, and sum of curvature geometries (all (*r*_s_ (18) ≥ 0.479, all *p*s ≤ 0.044). In addition, a negative correlation between ACC theta power and pure surface type and 2D rectangular geometries was observed (all *r*_s_ (18) ≤ −0.499, all *p*s ≤ 0.035). There was a positive correlation between arousal and theta power in ACC (*r*_s_ (18) = 0.720, *p* = 0.01). The same positive covariation was observed for pleasure ratings and modulations in the theta frequency range (*r*_s_ (18) = 0.573, *p* = 0.013).

Nonparametric partial correlation was computed to determine the relationship between form features and the ACC theta activity whilst controlling for arousal and pleasure. There was a positive significant partial correlation between surfaces with attachment type (*r*_s_ (14) = 0.525, *p* = 0.037) and a significant negative correlation between 2D rectangular geometries and theta power in the ACC (*r*_s_ (14) = −0.579, *p* = 0.019). The results indicate that arousal and pleasure had an influence on the relationship between theta power in the ACC and most of the form features. However, surfaces with attachment type and 2D rectangular geometries had a direct impact on theta activity in the ACC above and beyond valence and arousal.

## Discussion

This study investigated human brain dynamics related to the affective impact of interior forms when the perceiver actively explores an architectural space. The study was based on a formal clustering of architectural interior forms (Banaei et al., 2017) to provide a precise description of architectural forms and to bridge the gap with previous neuroarchitectural studies. As architectural design is concerned with users’ emotional experience, this study investigated the emotional experiences of different architectural forms in existing places. To allow the architectural experiences to be as realistic as possible, the participants actively moved inside the rooms, perceiving forms from different perspectives. To allow an investigation of the affective and cognitive processes and the accompanying brain dynamics during active perception of these different architectural forms, a mobile EEG setup synchronized to head-mounted VR was used.

Formal comparisons between rooms that were differently rated regarding valence and arousal revealed that rooms associated with lower pleasure and arousal ratings contained more linear geometries, while rooms with higher pleasure and arousal ratings contained more curvature geometries. The subjective measures were reflected in pronounced activity in or near the ACC for rooms that were rated higher in terms of pleasure and arousal. The formal evaluation of these rooms revealed higher densities of curvature geometries. Vartanian et al. (2013) showed similar results in an fMRI study, demonstrating higher activity in the ACC for beauty judgments of interior curvature contours. Other lines of evidence suggest a role of the ACC in emotional (Bush et al., 2000; Etkin et al., 2011), aesthetic, and artistic experiences (Kawabata and Zeki, 2004; Vartanian and Goel, 2004; Jacobsen et al., 2006; de Tommaso et al., 2008; Chatterjee, 2011). Using a mobile EEG setup synchronized to VR replicated increased ACC activity for curved features in architectural spaces. Importantly, the high temporal resolution of the EEG helped to describe the time course of the affective processing of architectural forms. This revealed a fast impact of the architectural space on activity in ACC starting around 50 ms.

Using precise form feature analysis allowed us to get deeper insights into ACC activity. Creating two groups of rooms with different features that differed in their emotional ratings, we observed significant differences in linear and curvature geometries that had an impact on theta activity generated in or near the ACC. Theta power correlated with curvature and 2D rectangular geometries, as well as the feature’s type (pure surfaces and surfaces with attachment) and Z-axis angles (sum of sloped, curvature, and combination Z-axis). Both arousal ratings and ACC activity revealed correlations with form features. While angle (XY-axis) and scale (object/object and object/context) correlated with arousal scores, these features did not show any significant correlation with theta activity in the ACC. The surface type (pure and surfaces with attachment) and 2D rectangular geometries that correlated with ACC activity were not associated with arousal scores. To control for the influence of affective processes on ACC activity (Bush et al., 2000; Etkin et al., 2011; Yu et al., 2011), a partial correlation controlling for arousal and pleasure ratings was computed. The results showed an impact of surfaces with attachment type and 2D rectangular geometries beyond the emotional impact of these features. Such surfaces with attachment could be walls, ceilings, or floors having additional feature(s) like a linear solid or a 3D solid attached to it. This finding indicated that the significant changes in ACC theta band activity was not only because of the effect of arousal or valence of the form features, but also because of the processing aspects of architectural features in the built environment.

Several studies revealed that affective responses to environments are automatic and unconscious (Ulrich, 1983, p. 91; Korpela et al., 2002; Valtchanov and Ellard, 2015; Coburn et al., 2017). This rapidly occurring effect beyond the conscious reflection of affective processes cannot be described without neuroscientific methods with high temporal resolution. While EEG showed the instantaneous effect of forms on brain dynamics while moving through architectural spaces, the SAM test allowed assessing the emotional impact after perceiving different perspectives of the rooms. Different covariations between electrophysiological activity and subjective ratings might thus be attributed to the delay in the subjective test at the end of a trial where the complete room was experienced for an extended period of time.

The ACC is involved in motor control, cognition and arousal (Paus, 2001). Aesthetic experiences, which represent a mixture of these processes, arise from neural systems underlying the sensory–motor, emotion–valuation and meaning–knowledge interactions (Chatterjee and Vartanian, 2014). The present study demonstrated comparable changes in theta activity originating in or near the ACC for two groups with different emotion ratings (containing movement, cognition, and arousal) and perception of different room perspectives (containing movement and cognition). These results further support the central role of ACC in aesthetic and architectural experiences. Because we inhabit and move in architectural space, our responses to architecture can be different from our responses to art (Pallasmaa, 2005, p. 63; Coburn et al., 2017). The aesthetic experience of both architecture and art, however, is reflected in the ACC activity. One possible functional explanation of the fast theta response originating in ACC might be the affordance of an architectural space that can be actively explored. As per the aesthetic triad of Chatterjee and Vartanian (2014), sensory-motor perception of rectilinear and curvature geometries can influence this aesthetic experience as well as differences in the perceived emotion and meaning. Along the same lines, Pallasmaa (2005) suggested the interaction between the sensory-motor and meaning (memory) to perceive architecture. He emphasized that architecture is not a series of images, nor are visual units and gestalt. Architecture is a kind of experience that interact with meaning (memory) and body movement (Pallasmaa, 2005, p. 63). During natural strolling, curves represent continuous forms and have similar views from different perspectives, while rectilinear geometries have focal points and provide different views from different perspectives.

The experience of different room perspectives was associated with differences in the density of form features and the time that the participants perceived these features while moving through the architectural space. The first impression with the onset of the room entrance gave a view of the ceiling, the floor and the walls of the space. After the onset of these stimuli, participants walked or turned and perceived different perspectives. Consequently, experiencing the right and front walls was based on fewer form features and only a limited view of the specifics of the room walls. The view into the complete space and the views toward specific walls had different perspectives. Additionally, while both room onset and front wall perspectives included the front wall, they differed in terms of the perceived depth of the space. Different room perspectives were accompanied by differences in ACC activity, implying a strong impact of the first impression of a room. This impression faded with the passing of time in the same architectural space, leading to repeated and comparable visual input. The significant impact of the first impression was reflected in the early onset of theta power increases of about 500 ms after the stimulus onset. Also within this time period, the differences in architectural features became apparent, as demonstrated in the significant differences in ACC theta modulations in curvature geometric forms.

While ACC activity showed a clear association with affective ratings and form features, other brain areas also demonstrated task-related activity. As expected, motor areas revealed significant differences in spectral perturbations between different perspectives of the rooms. This result revealed different activation patterns depending on the movement through space to get a different perspective for the same room. Spectral perturbations in motor areas in both the right and the left hemispheres were comparable for movement through architectural spaces. This result supports the assumption that exploration and the accompanying brain dynamics did not differ between different architectural interiors above and beyond active movement. Posterior areas of the brain (posterior cingulate cortex and occipital lobe) showed higher activity in the beta band when participants experienced the right wall, potentially reflecting changes in perspective during movement in space. The posterior cingulate cortex revealed significant differences between perspectives (room onset and right wall) and depth changes (room onset and front wall). While there is some evidence regarding the involvement of the PCC in emotional processes (Maddock, 1999; Maddock et al., 2003), the changes in PCC more likely reflect heading changes during movement in space (Gramann et al., 2010b; Chiu et al., 2012; Lin et al., 2015). There seems to be a fundamental contrast between the functions of the anterior and the posterior aspect of the cingulate cortex, suggesting an involvement of the anterior part in emotional control and the posterior in spatial orientation (Vogt et al., 1992). Our results support this assumption, thereby revealing effects of heading changes in the posterior cingulate cortex but no effects of emotional ratings. In line with this argument, the occipital lobe demonstrated an activity pattern that reflected changes in perspective and perceived room depths. This is in line with the primary and secondary visual cortices in the perception of perspective (Welchman et al., 2005). Mainly neurons in the visual cortex are sensitive to shapes and the orientation of lines during motion (Mallgrave, 2010, p. 142) possibly reflected in beta band activity. Emotional ratings revealed no impact on activity in the occipital lobe. The absence of significant activation differences in the precuneus, as reported by Vartanian et al. (2015), might be explained by the differences in the architectural spaces in the two studies. While high ceilings activated the visuospatial area in the left precuneus in the study of Vartanian et al. (2015), the present study controlled room heights.

### Limitations

While this study is novel with respect to the methods used, some limitations should be considered. One of the important issues for designing this study was the time a perceiver spent in a room to explore different interior aspects and to develop an affective response to the same. Sufficient time is needed to extract emotional experiences to the stimuli (Bekhtereva and Müller, 2015). However, the longer participants spent in one room, the fewer the room trials that can be collected, leading to non-optimal signal-to-noise ratios for EEG analyses. This study was intended to achieve a reasonable number of trials for EEG analyses while presenting a variety of architectural spaces that were formally described using a quantitative measure to derive formal clusters of the feature space. Future studies should increase the number of room trials by focusing on selected clusters as described in this study.

## Conclusion

To sum up, the results of the present study indicate that natural movement through the built environment leads to rapid responses in the ACC that reflect a first affective response to the architectural features of the environment. Curvature forms lead to stronger theta synchronization in the ACC and are correlated with higher positive ratings regarding the affective state of the participants. Moreover, partial correlations with the density of 2D rectangular geometries and the type of surfaces clearly indicated an involvement of the ACC in processing architectural features beyond their emotional impact. While the posterior cingulate cortex and the occipital lobe were involved in the perception of different perspectives and changes in room depths, there was no significant difference for the rather small perceptual changes in perspective between different walls of the same architectural space. This indicates a strong initial impression of the environment on the affective and perceptual aspects of the space with a fast decline in the impact of architectural features on affective and perceptual processes.

The present study extends existing research by controlling the presentation using a quantitative approach in order to analyze architectural features, and by allowing the perceiver to walk through the architectural space to gain different perspectives of the same room. The results demonstrate that interior form is defined not only by the geometry and by the features such as type, location, scale, and angle, but also by the way the inhabitant experiences this environment. This study also sheds a new light on the role of the ACC in the natural experience of architectural spaces. Natural movement is the best way to investigate the brain dynamics underlying natural perception and affective processes associated with perceiving a 3D environment. The insights gained from this kind of MoBI approach using fine-grained analyses of architectural forms and natural movement within virtual spaces while recording EEG activity provide fresh insights into neuroarchitecture. This interdisciplinary study showed the possibility of using MoBI and VR to systematically develop architectural studies.

## Author Contributions

MB conceptualized the research. KG and MB developed the experimental design and wrote the manuscript. JH was involved in experimental design. MB created the 3D models and the VR environment. MB recruited experimental participants, acquired and analyzed the data. KG supervised and participated in the data analysis. JH, AY and KG supervised the research.

## Conflict of Interest Statement

The authors declare that the research was conducted in the absence of any commercial or financial relationships that could be construed as a potential conflict of interest.

## Footnotes

^1^https://unity3d.com

^2^http://www.sccn.ucsd.edu/eeglab

^3^http://www.talairach.org/
